# Potency and safety analysis of hemp delta-9 products: the hemp vs. cannabis demarcation problem

**DOI:** 10.1186/s42238-023-00197-6

**Published:** 2023-07-26

**Authors:** Lee Johnson, Marc Malone, Erik Paulson, Josh Swider, David Marelius, Susan Andersen, Dominic Black

**Affiliations:** 1 CBD Oracle, 17291 Irvine Blvd, Tustin, CA 92780 USA; 2 Infinite Chemical Analysis Labs, 8312 Miramar Mall, San Diego, CA 92121 USA

**Keywords:** Hemp, Delta-9 THC, Farm Bill, Agriculture Improvement Act, Cannabinoid potency

## Abstract

**Background:**

Hemp-derived delta-9 tetrahydrocannabinol (∆^9^ THC) products are freely available for sale across much of the USA, but the federal legislation allowing their sale places only minimal requirements on companies. Products must contain no more than 0.3% ∆^9^ THC by dry weight, but no limit is placed on overall dosage and there is no requirement that products are tested. However, some states—such as Colorado—specifically prohibit products created by “chemically modifying” a natural hemp component.

**Methods:**

Fifty-three ∆^9^ THC products were ordered and submitted to InfiniteCAL laboratory for analysis. The lab analysis considered potency, the presence of impurities, and whether the ∆^9^ THC present was natural or converted from CBD. The presence of age verification, company-conducted testing, and warning labels was also considered.

**Results:**

While 96.2% of products were under the legal ∆9 THC limit, 66.0% differed from their stated dosage by more than 10%, and although 84.9% provided a lab report to customers, 71.1% of these did not check for impurities. Additionally, 49% of products converted CBD to THC to achieve their levels, and only 15.1% performed age verification at checkout.

**Conclusions:**

Despite some positive findings, the results show that hemp ∆^9^ THC companies offer inaccurately labeled products that contain more THC than would be allowed in adult-use states. This raises serious issues around consumer safety, and consent when consuming intoxicating products. Steps to boost accountability for companies must be considered by either the industry or lawmakers if intoxicating hemp products are to remain on the market safely.

**Supplementary Information:**

The online version contains supplementary material available at 10.1186/s42238-023-00197-6.

## Background

Delta-9 tetrahydrocannabinol (∆^9^ THC) is the primary psychoactive component of the *Cannabis sativa L.* plant (Cooper and Haney 2009), with other cannabinoids like cannabidiol (CBD) attracting attention for their therapeutic properties (Russo and McPartland 2003) in recent years (National Academies of Sciences, Engineering, and Medicine (NASEM), 2017). While both cannabinoids have medical applications, ∆^9^ THC has largely been associated with recreational use. Until 2012 (Conference and of State Legislatures (NCSL): State Medical Cannabis Laws 2022), the prohibition of the recreational use of cannabis in the USA made it essentially impossible to obtain legally, except through certain medical channels.

However, things changed when the 2018 Agriculture Improvement Act (a.k.a. the “Farm Bill”) made industrial hemp legal at the federal level (Agriculture Improvement Act of (US), 2018). The legislation allowed for an explosion of CBD products, but there were unintended consequences. The Farm Bill removed the cannabinoids in hemp from the definition of marijuana in the Controlled Substances Act and defined hemp as containing less than 0.3% ∆^9^ THC by dry weight (Johnson-Arbor and Smolinske 2022).

This allows non-intoxicating CBD oils, for example, to be sold freely. However, loopholes quickly emerged, such as ∆^8^ THC, another psychoactive compound much like ∆^9^ except with less potent and long-lasting effects (Kruger et al. 2022b) and less binding affinity for the CB_1_ receptor (Tagen and Klumpers 2022). Since it is a natural component of hemp, provided that products containing it have less than 0.3% ∆^9^ by dry weight, they can contain as much ∆^8^ as they want. Some states have taken action to stop the sale and distribution of ∆^8^ THC (Johnson-Arbor and Smolinske 2022), but new loopholes (for example, the increase in products with hexahydrocannabinol (Casati et al. 2022)) are identified more quickly than lawmakers can close them.

While ∆^8^ THC is present in negligible amounts in the *Cannabis* plant, virtually all products sold to consumers use ∆^8^ THC produced from CBD (Tagen and Klumpers 2022) by cyclization (the closure of a ring after an acid-catalyzed activation of a double bond) (Marzullo et al. 2020). This creates potential legal issues at the federal level (because it may render it “synthetic” THC), but the conversion process itself has also been a target of state-level legislation (CO Department of Public Health and Environment (DPHE): Re: Production and/or Use of Chemically Modified or Converted Industrial Hemp Cannabinoids 2021; Commonwealth of Massachusetts: Hemp in Massachusetts: Faqs 2022; SB 0788 (Md.) 2022).

Hemp ∆^9^ products were devised through a very simple application of the Farm Bill’s 0.3% by dry weight limit. A 10 g gummy can contain roughly 10 g × 0.3% = 0.03 g = 30 mg of ∆^9^ THC and still be within the legal limit. In contrast, intoxicating cannabis edibles in legal states like California and Colorado tend to contain just 5 mg or 10 mg ∆^9^ THC per serving (Brangham 2014; Romine 2019). As an unavoidable consequence of the law as it is currently written, intoxicating “hemp” ∆^9^ THC products are widely available in most states.

There are many potential issues with this; however, the biggest is the minimal regulations imposed on these “hemp” companies, especially in comparison to the regulations of legal cannabis markets. For instance, in California (Medicinal and Adult-Use Commercial CannabisRegulations (CA) 2023), each product must be lab tested (for cannabinoid potency, residual pesticides, foreign material, heavy metals, microbial impurities, mycotoxins, moisture content, and residual solvents) and packaging must be child-resistant, tamper-evident, and resealable, containing a cannabis universal symbol and numerous other pieces of information, such as a batch number and a full ingredient listing. These and similar regulations protect consumers in states with legal cannabis, but are not a requirement for hemp under the Farm Bill.

Since hemp ∆^9^ THC products are intoxicating, many people argue that they should meet similar standards to edibles in states like California and Colorado (Hemp and Roundtable: Delta-8 2021), and be subject to the same requirements for things like warning labels and child-safe packaging. As with ∆^8^ THC products, it is also possible that some of the ∆^9^ THC in hemp products is created through cyclization, and consequently may be impacted by existing state legislation.

This study aims to investigate the hemp ∆^9^ THC market with this in mind. In particular, we aim to determine whether companies remain within legal limits, whether the stated dosages are accurate, whether the ∆^9^ THC was produced by cyclization, and whether companies performed safety testing on products and made sufficient effort to prevent minors from purchasing them.

## Methods

### Sample size and product selection

For the lab study and market analysis, we first identified and purchased a selection of the most popular products online from different brands. To identify brands, Google searches for “hemp delta-9 thc edibles,” “hemp delta-9 thc tinctures,” “hemp delta-9 thc vapes,” “hemp delta-9 thc products,” “full spectrum CBD + THC product,” and “compliant delta-9 thc product” were performed, and the first 20 pages of results (200 search results total) were reviewed. The relevant commercial results were selected, excluding third-party lists of products and educational content. In the event this process led to a specific product, we navigated to the overall category page for hemp ∆^9^ THC products. We also searched Reddit, Instagram, and YouTube to identify brands that were missed by the Google search. Companies listed on CBD Oracle’s internal database of hemp companies were also manually searched for hemp ∆^9^ THC products. In total, we identified 89 brands currently selling hemp ∆^9^ THC products.

We estimated that this represents around 75% of the total hemp ∆^9^ THC market, as of April 2022. It is unlikely that the search strategy identified all brands, particularly local brands or those with no online presence. It was estimated, as a result of this and our overall familiarity with the market, that the strategy captured around 75% of the market. This estimate is imperfect by definition, because it cannot be precisely known how many brands exist beyond the boundaries of our online search. With this in mind, we estimate that there were 120 brands selling hemp ∆^9^ as of April 2022.

To select specific products for the lab analysis, companies with a TrustPilot rating lower than 4 or with a Better Business Bureau (BBB) grade below B were excluded, as were any companies which didn’t ship to California and any products that didn’t mention a specific dosage of THC. Companies were also ranked for popularity using the number of customer reviews on-site and followings on social media websites. We selected the ∆^9^ THC product from each company with the highest number of customer reviews. In some cases, we bought multiple products from the same manufacturer to cover more types of product.

We ordered a total of 53 products with a credit card and had them shipped to the CBD Oracle office in Tustin, CA. However, owing to the nature of the market, the majority of them were edibles. The products included gummies (38 products), tinctures (3), vape pens (1), cookies (2), brownies (1), chocolate (1), candies (3), beverages (3), and rice krispies (1). The vast majority sold some form of edibles (total 46), but we identified a few companies offering tinctures, some offering beverages, and one that offers a vape pen. The 53 products came from 48 different brands, which we estimate represented 40% of the total hemp ∆^9^ market, as of April 2022. Manufacturers came from multiple states: AZ, CA, CO, FL, GA, IN, MA, MI, MN, MO, NC, NJ, NV, NY, OR, TN, TX, and WI.

### Lab analysis

Products were collected for lab analysis directly from the office in their original, sealed packaging and were tested within 2 weeks of purchase to avoid degradation. The lab analyses were performed by InfiniteCAL, a California Department of Cannabis Control (DCC) and International Organization for Standardization/International Electrotechnical Commission (ISO/IEC 17025) accredited lab. All products were tested for potency, and 10 randomly selected products were tested for impurities, including pesticides, mycotoxins, residual solvents, microbial contamination, heavy metals, and foreign materials.

Potency analyses for the mass/mass percentage concentrations of cannabinoids (∆^9^-THC, ∆^8^-THC, CBD, tetrahydrocannabinolic acid [THCa], cannabidiolic acid [CBDa], cannabigerol [CBG], cannabigerolic acid [CBGa], cannabinol [CBN], tetrahydrocannabivarin [THCV], cannabidivarin [CBDV], and cannabichromene [CBC]) were performed using ultra-high-performance liquid chromatography coupled with a diode array detector (UHPLC-DAD), and concentrations are determined in relation to a calibration curve established based on certified reference materials.

∆^9^ THC was extracted from the gummies/edibles using one of two parallel validated procedures. The standard procedure is as follows: Solid edible samples are cryoground to a fine powder to ensure homogeneity. A subsample (3.0 g) is weighed in a 50-mL centrifuge tube containing steel balls. Forty milliliters of methanol is added to the tube and subsequently weighed to determine the exact volume of solution. Solutions are then sonicated in a 55 °C water bath then vortexed. Solid edibles should be reduced to a silt consistency, and therefore, it may be necessary to repeat and alternate sonication and vortexing steps. After the desired consistency is reached, samples are centrifuged for 3 min at 4200 rpm. A subsequent dilution step is performed with methanol in a separate 15-mL tube. Solutions are then filtered with a 0.22-um filter into a 2-mL autosampler vial.

For samples suspected or confirmed to contain gelatin (which includes samples that do not reach the desired consistency using the standard procedure), an alternate similar procedure mirrors the standard preparation with the following changes: a mixture of 50:50 water/methanol is used in place of methanol for the extraction step, and the dilution step uses acetonitrile instead of methanol and is also centrifuged for 3 min at 4200 rpm.

The measured potency depends on how well the THC was extracted from the products. However, InfiniteCAL has performed extensive validation on edible products, with both internal sample recovery experiments (using edibles spiked with known amounts of cannabinoids) as well as external blind proficiency tests, which have shown the extraction and analysis techniques used to be both thorough and robust. Validation data can be provided upon request.

Pesticide and mycotoxin levels were determined using a combination of gas chromatography triple quad mass spectrometry (GC MS/MS) and liquid chromatography triple quad mass spectrometry (LC-MS/MS). Concentrations of arsenic (As), cadmium (Cd), mercury (Hg), and lead (Pb) were determined using inductively coupled plasma mass spectrometry (ICP-MS). Analyses for heavy metals were conducted in kinetic energy discrimination (KED) mode, using helium (He) as the collision gas and argon (Ar) as the carrier gas.

Residual solvent and terpene analyses were performed using headspace gas chromatography single quad mass spectrometry (HS-GC-MS). Microbial analysis was performed using real-time polymerase chain reaction (qPCR), with aliquots taken from the batch being incubated for 24 h to allow microbial growth before testing. Moisture content was determined using a moisture analyzer, with loss of moisture from a pre-defined sample calculated gravimetrically. Finally, foreign material testing was performed visually, either unaided or with a microscope magnifier.

Full details of the methodology are available from InfiniteCAL (Swider and Marelius 2022).

### ∆^9^ THC conversion markers

Samples were analyzed to determine whether the ∆^9^ THC present was naturally occurring in the hemp plant or whether it was produced through conversion from CBD. While it is not possible to determine the source of the ∆^9^ THC with absolute certainty, there are several indicators that strongly suggest one of three sources: the ∆^9^ THC naturally produced by a hemp plant, ∆^9^ THC sourced from a cannabis plant and ∆^9^ THC resulting from a conversion from CBD. Additional file 1 contains more detail about the markers used and the underlying chemistry. Note that these analyses were only performed with 49/53 products, due to very low quantities of minor cannabinoids in 3 samples, which made identification of the source challenging, and the remaining sample contained no THC.

### *Trans:cis *∆^9^ THC ratio

Schafroth et. al. (Schafroth et al. 2021) found that while *cis*-∆^9^ THC was entirely absent from a high-THC Bedrocan cultivar, 28/31 (90.3%) of hemp plants had *trans*:*cis* ratios between 1.3:1 and 8:1. These observations suggest that the biosynthetic pathways to produce ∆^9^ THC in classical hemp strains are not stereospecific and produce both *trans*-∆^9^ and *cis*-∆^9^ THC, while high-THC cannabis strains have a strongly stereospecific pathway to produce the (-)-*trans*-∆^9^ THC. The delineation of the two strain types based on the presence/absence of *cis*-∆^9^ THC can therefore provide potential markers to identify the source of ∆^9^ THC.

**Fig. 1:**
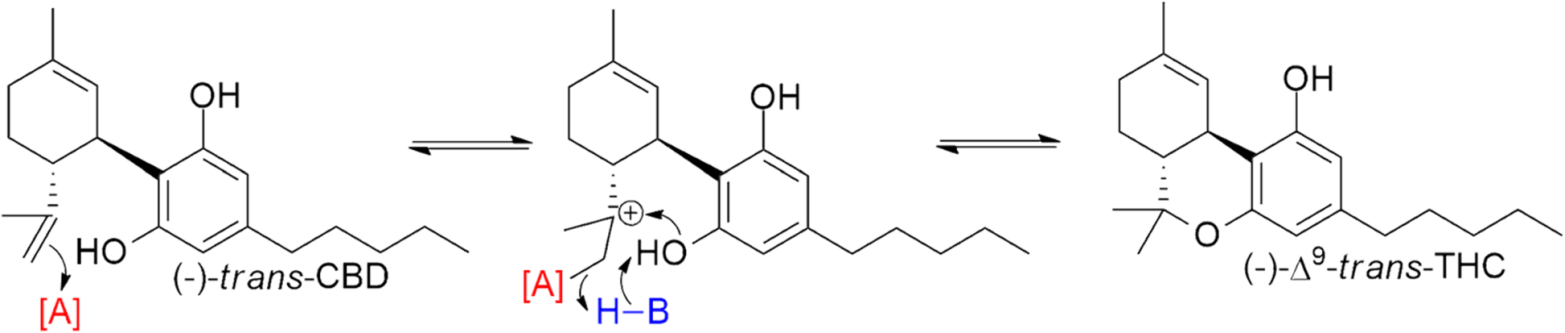
General scheme of conversion of (-)-trans-CBD to (-)-trans-∆9 THC

Since it is possible to synthetically form (-)-*trans*-∆^9^ THC directly from (-)-*trans*-CBD through an isomerization-free mechanism (Fig. 1), the ratios of *trans*:*cis*-∆^9^ in distillate converted from CBD can be expected to far exceed the ratios seen in natural hemp extracts. In contrast, oxidative cyclization from CBGa is the main source for biosynthesized THCa (Taura et al. 2007), and while natural conversion from CBD could theoretically produce *cis*-∆^9^ THC, this process would also lead to substantial amounts of ∆^8^ and ∆^10^ THC in *Cannabis* plants (Golombek et al. 2020), which is not observed. Therefore, in this analysis, *trans*:*cis* ratios > 8:1 are taken as evidence that the source of the THC is not hemp. If there is no *cis-*∆^9^ in the sample, it is likely the THC is sourced from cannabis.

### The quantity of ∆^8^THC and ∆^8^-iso-THC

∆^8^ THC occurs naturally in *Cannabis sativa L.*, but only in negligible amounts (Tagen and Klumpers 2022). ∆^8^ THC is formed during conversion from CBD to ∆^9^ THC (Marzullo et al. 2020), as is ∆^8^-*iso*-THC (along with its isomerized partner ∆^4(8)^-iso-THC). This “miscyclization” only presents itself in conversion reactions. This means that products created using naturally sourced ∆^9^ THC should have little to no ∆^8^ THC, with >1% ∆^8^-*iso*-THC+∆^8^ THC (relative to the ∆^9^ THC amount) being taken as evidence of conversion from CBD via cyclization (Marzullo et al. 2020).

Delineation of the ∆^8^-iso-THC and ∆^8^-THC amounts was not performed for this study, as the samples were run under standard UHPLC-DAD conditions for quantitation. The reported amounts of ∆^8^-THC, therefore, represent the combined contributions of both compounds.

### The quantity of cannabigerol

The presence and quantity of cannabigerol (CBG) and other minor cannabinoids were also used as indicators of the source of the ∆^9^ THC. Just as the ∆^8^ THC in commercial products is produced via cyclization from CBD (Tagen and Klumpers 2022) because levels in “hemp” (i.e., *Cannabis sativa L.* plants with less than 0.3% ∆^9^ THC) are not naturally high enough to have a psychoactive effect, the low ∆^9^ THC levels in many hemp plants (Glivar et al. 2020; Johnson and Wallace 2021; Schafroth et al. 2021) may encourage manufacturers to use the same approach. Since CBD is the starting point for the cyclization reactions, the most efficient starting material is high-purity CBD “isolate,” which is not likely to have significant amounts of CBG present.

However, while natural hemp (Glivar et al. 2020; Schafroth et al. 2021) and cannabis (Radwan et al. 2021) contain minor cannabinoids such as CBG, this is not a product of the cyclization reaction (Tagen and Klumpers 2022). If CBG is not present in the starting material nor a product of the conversion, it would not be expected to be present in converted ∆^9^ THC. Therefore, products with low quantities of CBG (<1% of total ∆^9^ THC content) are more likely to use converted ∆^9^ THC and those with higher quantities are more likely to use naturally sourced ∆^9^ THC.

Exact translation of fixed metrics for the ∆^9^-THC products in the study was not possible due to the wide range of ∆^9^-THC quantity in each product, but using the relative amounts of the three components along with some judgment calls allowed for grouping of each product into the three categories with reasonable confidence.

### Age verification checks

Since all products were purchased from the companies’ websites, their use of age verification measures was considered. For each product, we noted if they required an ID to be presented on purchase or if an easily-circumvented method (e.g., simply entering a birth date) (Williams et al. 2020) was used. Additionally, we also noted how many products required an adult signature on delivery.

### Packaging and labeling

The 53 products considered were inspected for warning labels, batch IDs, child-resistant containers, and the cannabis universal symbol (intended to alert consumers that the product contains large amounts of THC).

We define a warning label as a clear statement on the packaging that the product is intoxicating, is dangerous to minors and pets, or identifying situations in which you should not use the product, such as:

- For adults 18+, or 21+ where state law applies
- Will intoxicate, use extreme caution
- Keep away from children or pets
- Don’t drive after using
- Don’t operate heavy machinery
- Don’t consume if pregnant or breastfeeding
- Don’t consume if you are subject to drug testing
- Consult with your physician before use

Batch IDs are unique codes which identify a specific production batch of a product, thus enabling identification of other affected products in the event of contamination. These can have numerous formats, with an example from a purchased product being “E21362-10HC.”

Child-resistant containers are defined in law (Standards and (US) 1973) as those which 85% of children aged 3 to 5 cannot open within 5 min without a demonstration and which 80% still cannot open even after a demonstration. For example, a child-resistant cap may require the user to push down and twist the cap to open, rather than simply twisting. We did not test the packaging first-hand or verify that it met the legal definition, but took the presence of child-proofing mechanisms (such as a child-resistant cap) as evidence of a child-resistant container.

**Fig. 2:**
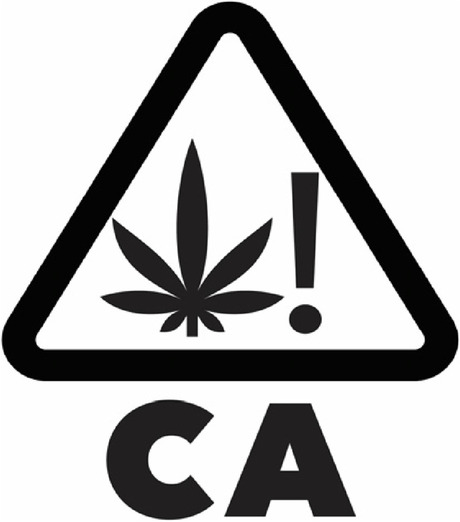
The cannabis universal symbol as used in California

The cannabis universal symbol (Fig. 2) or some variation thereof is used to signify to consumers that the product contains cannabis and may be intoxicating. This is not required under the 2018 Farm Bill (Agriculture Improvement Act of (US) 2018), but is required for high-THC products in adult-use markets such as California (California Department of Cannabis Control: Requirements for Cannabis Goods 2022).

### Lab reports provided by companies

In most cases, hemp consumers must depend on the Certificate of (lab) Analysis (COA) provided by the company itself to determine the true potency and safety of the product in question. These were also analyzed, in particular, whether they were tested for impurities or just potency, whether the lab used was ISO accredited, and whether they were DEA certified.

## Results

### Advertised and measured ∆^9^ THC potencies vs.adult-use states’ standard dosage

The products considered advertised between 0.5 and 40 mg of ∆^9^ THC per serving, with an average of 12.98 mg per serving across all products. Even the average figure is higher than the limit of 10 mg ∆^9^ THC placed on edibles in legal states such as Colorado and California, and the highest exceeds it by a factor of four. The mode of the dataset was 10 mg, but many products go beyond this limit. Based on lab-measured potencies, the mean was 10.08 mg, which is still slightly above the limit for most legal states. The highest lab-measured potency was 36.68 mg/serving, almost 3.7-fold higher than most adult-use limits.

### Potencies of other cannabinoids

The products sampled also contained small amounts of other cannabinoids. On average, the products contained 0.88% CBD, 0.026% CBC, 0.024% CBG, 0.004% CBN, and 0.03% ∆^8^ THC.

### Legality of commercial hemp ∆^9 ^THC products

Industrial hemp products are considered legal if they contain less than 0.3% ∆^9^ THC by dry weight. Of the 53 products analyzed, 96.2% (51 products) fell within the legal limit for ∆^9^ THC. The 2 products that exceeded the limit were the Blueberry Citrus Burst gummies from Delta Extrax (0.419% ∆^9^ THC) and the Straw Blasted gummies from Hixotic (0.31% ∆^9^ THC). The average for all products was 0.154% ∆^9^ THC, showing clearly that in many cases, the products are not even close to the legal limit. Overall, the vast majority of the hemp ∆^9^ THC products likely fall within limits specified by the Farm Bill.

### Comparison of advertised and measured ∆^9 ^THC potency

**Fig. 3:**
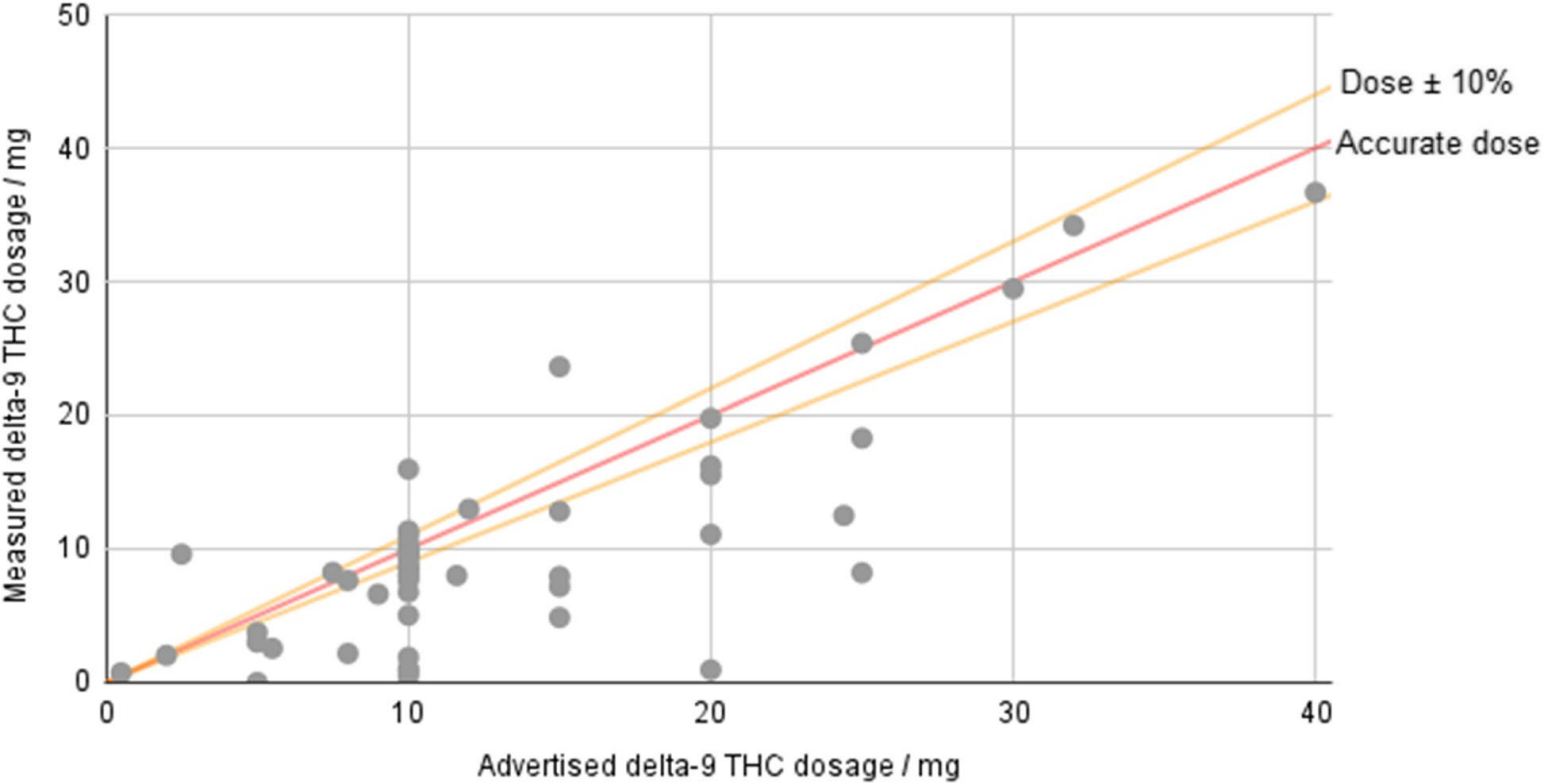
Measured vs. advertised dose in hemp ∆9 THC products

The measured amounts of ∆^9^ THC were compared with the amounts listed on the website and/or product packaging. Considering anything within 10% of the stated potency to be “accurately labeled,” the results show that 66.0% (35/53) of products were not accurately labeled. For all of the products, the average measured potency was 82.4% of the advertised amount (i.e., the mean of [measured potency/advertised potency] × 100 % = 82.4%). For the inaccurately labeled products, the average was 73.3% of the advertised amount. Overall, 88.6% of the mislabeled products contained less ∆^9^ THC than advertised (Fig. 3).

### Impurities in ∆^9 ^THC products

No pesticide residue, solvent residue, heavy metal contamination, microbial contamination, mycotoxins, or foreign matter were detected in any of the 10 products that were tested.

### *Trans:cis *∆^9^ THC ratios

The ratio of *trans* to *cis* ∆^9^ THC was used to determine whether the product likely used THC either sourced from cannabis or converted via cyclization, with hemp plants generally having a ratio of 8:1 or lower (Schafroth et al. 2021). The analysis showed that 77.6% (38/49) of products had ratios above the range expected for hemp plants.

### ∆^8^ THC + ∆^8^-iso-THC content of products

With only negligible quantities of ∆^8^ THC in natural hemp or cannabis plants, products with ∆^8^-*iso*-THC + ∆^8^ THC at levels >1% of the total ∆^9^ THC content likely involved some THC production from cyclization. A total 63.3% (31/49) of products contained more than 1% ∆^8^ THC + ∆^8^-*iso*-THC.

### CBG content of products

Minor cannabinoids such as CBG are commonly found in products sourced from hemp or cannabis, but are almost entirely absent from products produced via cyclization. In the analysis, 38.8% (19/49) of products contained less than 1% CBG (relative to total ∆^9^ THC content) and thus were less likely to be made using natural cannabis or hemp extract.

### Naturally occurring vs. converted ∆^9^ THC

Based on the lab analysis and specifically the factors discussed above, InfiniteCAL estimated that 49.0% (24/49) of products used ∆^9^ THC that had been converted from CBD through cyclization. The results also suggested that 26.5% (13/49) used ∆^9^ THC sourced from cannabis to meet their stated dosage and that only 18.4% (9/49) probably used natural hemp-derived ∆^9^ THC. The remaining four products studied could not be classified.

### Analyzing the COAs provided by hemp ∆^9^ companies

84.9% (45/53) of the hemp ∆^9^ THC products investigated had an associated COA available to customers. Of these products with COAs, 71.1% (32/45) were not tested for impurities, with tests conducted only to verify potency. In total, then, 75.5% (40/53) of products were not proven to be free from impurities (although testing a random sample of 10 revealed no issues, as discussed above).

80% (36/45) of COAs were obtained from ISO/IEC 17025:2017 certified labs, and accreditation certificates were verified through PJLA, A2LA, and ILAC databases. In addition, 51.1% (23/45) of COAs were from labs that were DEA certified.

### Labeling of hemp ∆^9^ THC products

Customers depend on labels for important information and warnings about the contents of a product. It was found that 83.0% (44/53) of products had some form of warning label.

However, 73.6% (39/53) of products did not include the cannabis universal symbol (or some variation thereof) on their packaging. In adult-use states, this is required to inform customers that the product contains large amounts of THC (California Department of Cannabis Control: Requirements for Cannabis Goods 2022). Additionally, 50.9% (27/53) of products did not include a batch ID on the label, which means it will be difficult or even impossible to trace any issues back to a specific batch and inform consumers and retailers of the issue.

### Age verification and child-resistant packaging for hemp ∆^9^ THC products

The products we sampled largely did not include child-resistant packaging, with 81.1% (43/53) not using a child-resistant container. This would be illegal in adult-use markets such as California (California Department of Cannabis Control: Requirements for Cannabis Goods 2022), for instance.

For age verification, 84.9% (45/53) of companies did not perform online age verification at checkout. The companies which did all used AgeChecker, which verifies age by cell phone verification and requiring customers to upload a “selfie” with their ID, as well as provide a clear picture of it. Additionally, 98.1% of companies (52/53) did not require an adult signature on delivery. All but one product was simply left in the mailbox without obtaining any form of signature.

## Discussion

The lab analysis revealed that the industry offers what it claims in some ways, but potentially misleads or does not inform customers in others. On the one hand, it is clear that the vast majority of products (96.2%) fall within the legal ∆^9^ THC limits established by the 2018 federal Farm Bill (Agriculture Improvement Act of (US) 2018). Additionally, the tests conducted suggest that impurities are not a substantial issue for hemp ∆^9^ THC companies.

However, the investigation also revealed several points which may be concerning from a consumer perspective. Firstly, in many cases, the advertised dosages are substantially higher than would be allowed in any regulated, legal, high-THC cannabis market in the country. Secondly, most of the products (73.6%) do not include the cannabis universal symbol to warn potential consumers of their high ∆^9^ THC content. Despite the fact that most (84.9%) products are backed with a COA, since most of these do not include impurity testing, 75.5% of hemp ∆^9^ THC products are not safety tested. This problem is compounded by the lack of batch labeling for 50.9% of products. Finally, and potentially the largest issue for consumers is the fact that 66.0% of products differ from the ∆^9^ THC dosage stated on their labels by 10% or more, usually providing less than advertised.

Considering the potential risks of excessive amounts of THC (D’Souza et al. 2009), the combination of factors here could be a cause for concern. Customers in adult-use states expect the cannabis universal symbol or some variation thereof on high-THC products, with over 80% of states with medical cannabis requiring this, for instance (Kruger et al. 2022a). In many cases, even the advertised dosages exceed those considered acceptable in adult-use states. Additionally, two out of three products differ from the stated dose by over 10%, which makes it difficult—if not impossible—for customers to know how much THC they will actually consume. The problem from a consumer perspective is one of consent: people may buy under the assumption that the product is “just hemp” or at least not high-THC, and others may get more THC than they wanted or were informed of through labeling. These issues could be solved by the industry, but legislators might also take steps such as setting a total THC cap on hemp products or requiring accurate labeling.

Despite some concern about youth access to intoxicating hemp products (Akingbasote et al. 2022; FDA: Accidental Ingestion by Children of Food Products Containing THC 2022), the analysis revealed many problems with how companies attempt to prevent under-age purchases and access. In particular, only 8 of 53 products (15.1%) required any form of age verification prior to the order being placed. This means that in the remainder of cases, all people had to do prior to making a purchase is either input a date of birth or click a button confirming that he/she is over 21. These systems can be easily circumvented or simply lied to (Williams et al. 2020) and so do not constitute age verification. The remaining purchases had age verification through AgeChecker, which uses public records and images of ID to determine the customer’s true age. Additionally, only 1 product (1.9%) required an adult signature on delivery, and the remainder simply left the delivery in the mailbox.

While getting a valid card to pay may be a challenge, this is essentially the only thing preventing youths from accessing high-THC hemp products such as those in this study. From this point onwards, he or she could easily place an order (simply using an alternative website if they initially chose one of the 15% which performs age checks) and receive the delivery without having to present ID at any stage. In the absence of further laws—requiring a signature on delivery for intoxicating hemp products, for instance—this situation is difficult to rectify.

Finally, the lab analysis showed that 96.2% of hemp ∆^9^ THC products do fall within the limits established by the Farm Bill. This is a positive sign for the industry, noting that current legal opinion suggests that hemp ∆^9^ THC is legal in the absence of further state legislation. However, the finding that 49% of products used ∆^9^ THC converted from CBD may challenge this in some localities. Colorado (CO Department of Public Health and Environment (DPHE): Re 2021), for example, restricts ∆^8^ THC on the basis that, “chemically modifying or converting any naturally occurring cannabinoids from industrial hemp is non-compliant with the statutory definition of ‘industrial hemp product,” and Massachusetts (Commonwealth of Massachusetts: Hemp in Massachusetts: Faqs 2022) has a similar approach. These laws would also apply to ∆^9^ THC products advertised as hemp if levels were increased by conversion from CBD. Maryland is also considering (SB 0788 (Md.) 2022) regulations on THC products that are synthetically or artificially derived.

There are some limitations of the analysis. Firstly, owing to the nature of the market, the majority of products (86.8%) tested were gummies, candies, or other edibles, with relatively few drinks, tinctures, and vaping products. While this represents the market closely, it may be that other product types differ in some important ways (for example, being more likely to exceed the legal limit for ∆^9^). Unfortunately, the sample did not include enough non-edible samples to investigate differences by product type. Only one sample of each product was analyzed, but values could (and likely do) vary by the individual sample and not just by product. There was also some time (less than 2 weeks in all cases) between purchase and testing, which could feasibly have impacted the results through degradation, despite all products being sealed until testing.

The results show the consequences of the legal loophole which hemp ∆^9^ THC companies are currently operating in. With no centralized regulatory body, and very little in the way of state-specific regulations in most cases, intoxicating hemp products are currently allowed to operate with minimal oversight. This is why, for instance, that not all products are accompanied by a COA, why dosages (both advertised and actual) vary so wildly, and why in most cases age verification is essentially absent.

There are three avenues that could help companies and regulators find a potential solution to these issues. Firstly, states with adult-use or medical marijuana programs could incorporate intoxicating hemp products into their existing legal framework for legal cannabis. This would include, for instance, maximum doses of ∆^9^ THC (or indeed other THCs) per serving and per package, as well as lab testing requirements. Secondly, states could improve their hemp legislation to account for intoxicating products, enabling them to implement similar restrictions on THC-rich products and require age verification. Finally, the industry itself could adopt reasonable standards in the absence of official guidance. In the manner most companies now offer COAs for their products, they may (through hemp industry organizations) institute mandatory age checks and standardized testing requirements.

## Conclusion

The legal status of hemp ∆^9^ THC products in America essentially permits their open sale while placing very little requirements on the companies selling them. The results of this lab and market analysis show the consequences of this policy: products are sold that have 3.7 times the THC content of edibles in adult-use states, and age verification, safety testing, and accurate dosages are neither required nor often present. On top of this, 49% of products use THC converted from CBD, which explicitly contradicts the law in some states but raises issues with the Farm Bill’s definition of “hemp” in any case. The industry meets some common-sense requirements, such as almost all products being within legal limits and none having issues with impurities, but there is also a lot of work to do in several key areas.

### Supplementary Information

**Additional file 1:** (Kramer M, Lomas S, 2017).

## Data Availability

The original lab reports for each product are available in a Google Drive.

## Abbreviations

CBC: Cannabichromene
CBD: Cannabidiol
CBDa: Cannabidiolic acid
CBDV: Cannabidivarin
CBG: Cannabigerol
CBGa: Cannabigerolic acid
CBN: Cannabinol
COA: Certificate of Analysis
DCC: Department of Cannabis Control
DEA: Drug Enforcement Administration
GC MS/MS: Gas chromatography triple quad mass spectrometry
HPLC: High-performance liquid chromatography
HS-GC-MS: Headspace gas chromatography single quad mass spectrometry
ICP-MS: Inductively coupled plasma mass spectrometry
ISO/IEC: International Organization for Standardization / International Electrotechnical Commission
KED: Kinetic energy discrimination
LC-MS/MS: Liquid chromatography triple quad mass spectrometry
qPCR: Real-time polymerase chain reaction
THC: Tetrahydrocannabinol
THCa: Tetrahydrocannabinolic acid
THCV: Tetrahydrocannabivarin
UHPLC-DAD: Ultra-high-performance liquid chromatography coupled with a diode array detector
UV: Ultraviolet

## References

[CR1] Agriculture Improvement Act of 2018 (US).

[CR2] Akingbasote J, Szlapinski S, Charrette A, Hilmas C, Guthrie N. Safety of cannabis- and hemp-derived constituents in reproduction and development. In: Reproductive and Developmental Toxicology, 3rd ed. Academic Press; 2022. pp.455-487. https://www.sciencedirect.com/science/article/pii/B9780323897730000242 Accessed 17 June 2023.

[CR3] Brangham W. Edible Marijuana Rules Tightened in Colorado. PBS NewsHour. 2014.

[CR4] California Department of Cannabis Control: Requirements for Cannabis Goods. 2022.

[CR5] Casati S, Rota P, Bergamaschi RF, Palmisano E, La Rocca P, Ravelli A, Angeli I, Minoli M, Roda G, Orioli M. Hexahydrocannabinol on the Light Cannabis Market: The Latest ‘New’ Entry. Cannabis Cannabinoid Res*. *2022. 10.1089/can.2022.0253 Accessed 17 June 2023. 10.1089/can.2022.0253 36445181

[CR6] Commonwealth of Massachusetts: Hemp in Massachusetts: Faqs. 2022. https://www.mass.gov/guides/hemp-in-massachusetts-faqs#-is-it-legal-to-manufacture-delta-8-thc-from-hemp?-. Accessed 17 June 2023.

[CR7] CO Department of Public Health and Environment (DPHE): Re: Production and/or Use of Chemically Modified or Converted Industrial Hemp Cannabinoids. 2021.

[CR8] Cooper ZD, Haney M (2009). Actions of delta-9-tetrahydrocannabinol in cannabis: relation to use, abuse, dependence. Int Rev Psychiatry.

[CR9] D’Souza DC, Sewell RA, Ranganathan M (2009). Cannabis and psychosis/schizophrenia: human studies. Eur Arch Psychiatry Clin Neurosci.

[CR10] FDA: Accidental Ingestion by Children of Food Products Containing THC. 2022.

[CR11] Glivar T, Eržen J, Kreft S, Zagožen M, Čerenak A, Čeh B, Tavčar Benković E (2020). Cannabinoid content in industrial hemp (cannabis sativa L.) varieties grown in Slovenia. Ind Crops Prod.

[CR12] Golombek P, Müller M, Barthlott I, Sproll C, Lachenmeier DW (2020). Conversion of cannabidiol (CBD) into psychotropic cannabinoids including tetrahydrocannabinol (THC): A controversy in the scientific literature. Toxics.

[CR13] Johnson MS, Wallace JG (2021). Genomic and chemical diversity of commercially available high-CBD industrial hemp accessions. Front Genet.

[CR14] Johnson-Arbor K, Smolinske S (2022). The current state of delta-8 THC. Am. J. Emerg. Med..

[CR15] Kramer M, and Lomas S. Separation of Twelve Major Cannabinoids using a Luna Omega Polar C18 in Two Particle Sizes: 3 μm and 5 μm. 2017. https://phenomenex.blob.core.windows.net/documents/9323f9ab-c5ed-4958-bb05-c03f34590cc6.pdf Accessed 17 June 2023.

[CR16] Kruger D, Korach N, Kruger J (2022). Requirements for Cannabis Product Labeling by U.S. State. Cannabis Cannabinoid Res.

[CR17] Kruger JS, Kruger DJ (2022). Delta-8-THC: Delta-9-THC’s nicer younger sibling?. J Cannabis Res.

[CR18] Marzullo P, Foschi F, Coppini DA, Fanchini F, Magnani L, Rusconi S, Luzzani M, Passarella D (2020). Cannabidiol as the Substrate in Acid-Catalyzed Intramolecular Cyclization. J Nat Prod.

[CR19] Medicinal and Adult-Use Commercial Cannabis Regulations 2023 (CA) https://cannabis.ca.gov/wp-content/uploads/sites/2/2023/02/dcc_commercial-cannabis-regulations_2023-0131.pdf Accessed 17 June 2023.

[CR20] National Academies of Sciences, Engineering, and Medicine (NASEM). Therapeutic effects of cannabis and cannabinoids. In: The Health Effects of Cannabis and Cannabinoids: The Current State of Evidence and Recommendations for Research. Washington (DC): National Academies Press (US); 2017. https://www.ncbi.nlm.nih.gov/books/NBK425767/. Accessed 17 June 2023. 28182367

[CR21] National Conference of State Legislatures (NCSL): State Medical Cannabis Laws. 2022.

[CR22] Poison Prevention Packaging Standards 1973 (US).

[CR23] Radwan MM, Chandra S, Gul S, ElSohly MA (2021). Cannabinoids, phenolics, terpenes and alkaloids of cannabis. Molecules.

[CR24] Romine S. Before You Buy Cannabis, Brush up on California Laws and Safety Precautions. USA Today. 2019.

[CR25] Russo EB, McPartland JM (2003). Cannabis is more than simply delta(9)-tetrahydrocannabinol. Psychopharmacology.

[CR26] SB 0788 2022 (Md.). https://mgaleg.maryland.gov/mgawebsite/Legislation/Details/sb0788 Accessed 17 June 2023.

[CR27] Schafroth MA, Mazzoccanti G, Reynoso-Moreno I, Erni R, Pollastro F, Caprioglio D, Botta B, Allegrone G, Grassi G, Chicca A, Gasparrini F, Gertsch J, Carreira EM, Appendino G (2021). Δ9-cis-Tetrahydrocannabinol: Natural Occurrence, Chirality, and Pharmacology. J Nat Prod.

[CR28] Swider J, and Marelius D. InfiniteCAL Certificate of Methodology. 2022. https://infinitecal.com/wp-content/uploads/2022/12/InfiniteCAL-Certificate-of-Methodology-2022.pdf Accessed 17 June 2023.

[CR29] Tagen M, Klumpers L (2022). EReview of delta-8-tetrahydrocannabinol (Δ8-THC): Comparative Pharmacology with Δ9-THC. Br J Pharmacol.

[CR30] Taura F, Sirikantaramas S, Shoyama Y, Shoyama Y, Morimoto S (2007). Phytocannabinoids In cannabis sativa: recent studies on biosynthetic enzymes. Chem Biodivers.

[CR31] US Hemp Roundtable: Delta-8. 2021. https://hempsupporter.com/news/delta-8. Accessed 17 June 2023.

[CR32] Williams RS, Phillips-Weiner KJ, Vincus AA (2020). Age Verification and Online Sales of Little Cigars and Cigarillos to Minors. Tob Regul Sci.

