# Correction: Potency and safety analysis of hemp delta-9 products: the hemp vs. cannabis demarcation problem

**DOI:** 10.1186/s42238-023-00201-z

**Published:** 2023-08-05

**Authors:** Lee Johnson, Marc Malone, Erik Paulson, Josh Swider, David Marelius, Susan Andersen, Dominic Black

**Affiliations:** 1 CBD Oracle, 17291 Irvine Blvd, Tustin, CA 92780 USA; 2 Infnite Chemical Analysis Labs, 8312 Miramar Mall, San Diego, CA 92121 USA

**Correction: J Cannabis Res 5, 29 (2023)**

**https://doi.org/10.1186/s42238-023-00197-6**

Following publication of the original article (Johnson et al. 2023), a typographical error has been reported in the 6th authors’ name. “Susan Anderson” should read “Susan Andersen”.

This has been corrected above and the original article has been updated.
