# Supplementary material for: Potency and safety analysis of hemp delta-9 products: the hemp vs. cannabis demarcation problem
Source: J Cannabis Res. 2023 Jul 26;5:29. doi: 10.1186/s42238-023-00197-6 (PMC10369762; doi:10.1186/s42238-023-00197-6)
Supplement: Supplementary file 1 — Additional file 1: (Kramer M, Lomas S, 2017). [file 42238_2023_197_MOESM1_ESM.docx]

**Supplementary Information**

∆^9^ **THC Conversion Markers**

Samples were analyzed to determine whether the ∆^9^ THC present was naturally-occurring in the hemp plant or whether it was produced through conversion from CBD. In an effort to label the plants by their genetic disposition rather than their absolute THC content, in this section, the terms ‘cannabis’ and ‘hemp’ distinguish the cultivar groupings by their THC-to-CBD ratio: ‘cannabis’ refers to strains of *Cannabis sativa L.* that are cultivated for their THC content and produce a high THC-to-CBD ratio (>10:1), and ‘hemp’ refers to strains that preferentially produce CBD with a low THC-to-CBD ratio (<1:10).


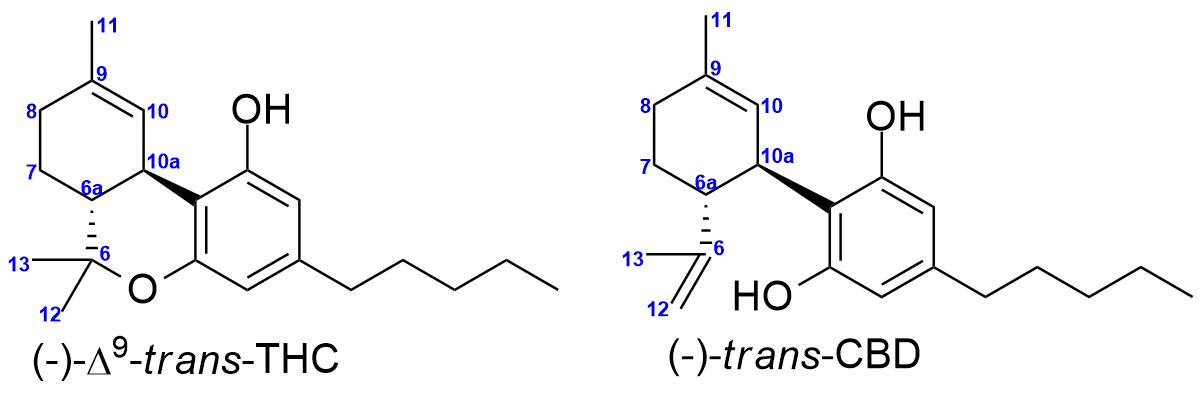


**Figure 4.** Carbon numbering system for THC and CBD

The structure of ∆^9^-THC contains two chiral centers at positions labeled 6a and 10a (Figure 4). The two chiral centers present four possible stereoisomers for the compound (Figure 5). The (+)-*cis* and (-) *cis*-∆^9^ isomers are diastereomeric from the two *trans* isomers, allowing for facile chromatographic separation using traditional HPLC columns [Kramer and Lomas, 2017]. Verification of the *cis*-∆^9^ THC signal is achieved through a comparison of retention time and ultraviolet (UV) profile to authentic (±)-*cis*-∆^9^ THC available from Cayman Chemical (Product # 35012). 


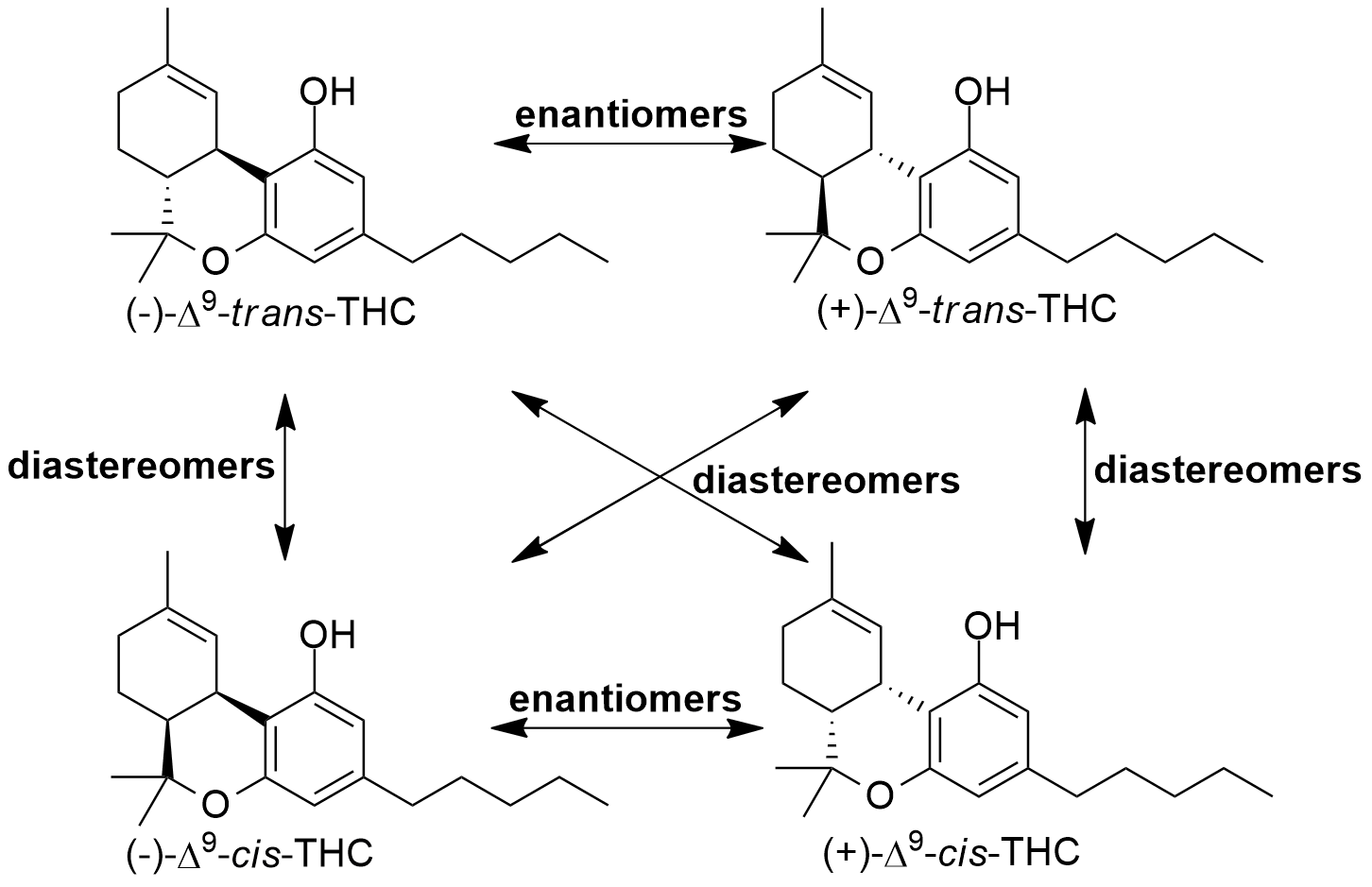


**Figure 5.** Stereochemical relationships between isomers of ∆-THC

In a detailed analysis of distillate/isolate samples received at InfiniteCAL from July to October 2021 (unpublished observations from Erik Paulson, Josh Swider and David Marelius), distinct differences were seen between samples extracted from hemp biomass as opposed to cannabis extract. Despite the high levels of *trans*-∆^9^ tetrahydrocannabinol (typically >70%), *little to no cis-*∆^9^ *tetrahydrocannabinol (<0.1%) was found in virtually all distillate sourced from cannabis.* Conversely, even though the levels of *trans*-∆^9^ THC were considerably lower in hemp extracts (0.1-10% *trans*-∆^9^), levels of (±)-*cis*-∆^9^ THC typically exceeded 0.5% (Note: Since the (+)-*cis*-∆^9^ THC and (-)-*cis*-∆^9^ THC enantiomers coelute during typical (non-chiral) chromatography, the assignment of the signal to one of the two is not possible, so the signal must be represented as (±)-*cis*-∆^9^ THC until further information is available). Importantly, the ratios between *trans*- and *cis*-∆^9^ signals in hemp extract generally ranged between 2:1 to 5:1.  

The InfiniteCAL analysis of hemp and cannabis extracts agreed with observations by Schafroth *et. al.* [19], who found that no *cis*-∆^9^ THC was found in a high-THC Bedrocan cultivar, while 27 of 31 hemp cultivars tested produced *trans*:*cis* ratios between 1.3:1 to 6:1. The InfiniteCAL and Schafroth observations suggest that the biosynthetic pathways to produce ∆^9^ THC in classical hemp strains are not stereospecific and produce both *trans*-∆^9^ and *cis*-∆^9^ THC, while high-THC cannabis strains have a strongly stereospecific pathway to produce the (-)-*trans*-∆^9^ THC. The delineation of the two strain types based on the presence/absence of *cis*-∆^9^ THC can therefore provide potential markers to identify the source of ∆^9^ THC.


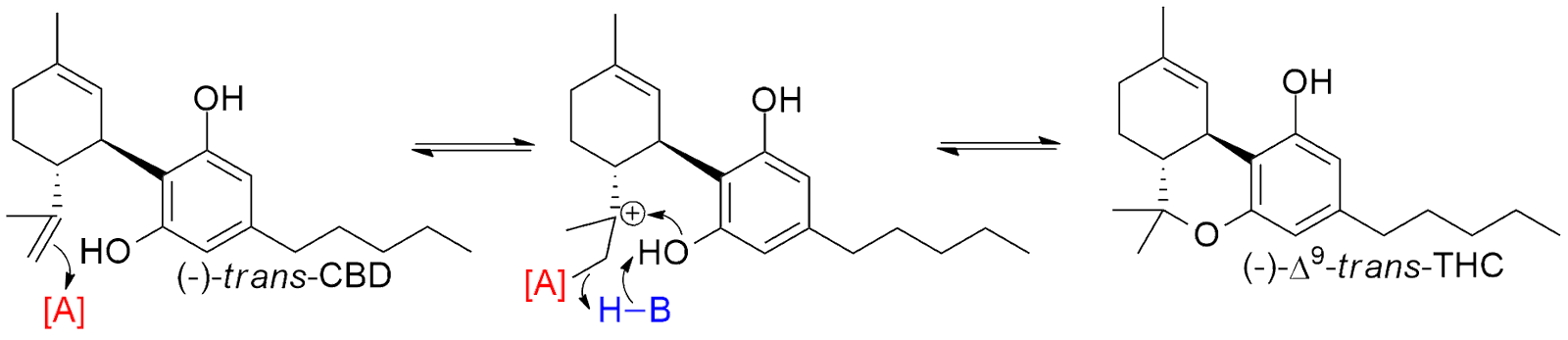


**Figure 1.** General scheme of conversion of (-)-*trans*-CBD to (-)-*trans*-∆^9^ THC

The increasing prevalence of the use of chemical synthesis to produce ∆^9^ THC from CBD adds an extra layer of complexity to the use of *cis*-∆^9^ as a marker. The ideal conversion of CBD to ∆^9^ THC requires only a protonation of the double bond between carbons 6 and 12, followed by an attack of one of the two adjacent -OH groups to cyclize the ring. Such a cyclization would convert the predominant form of CBD ((-)-*trans* CBD) exclusively to the (-)-*trans*-∆^9^ THC isomer (Figure 1, reproduced from main text). However, we observed that samples that were confirmed to us to be converted samples also had elevated levels of (±)-cis-∆^9^, generally between 0.25-1%. While it is possible that the *cis*-∆^9^ THC could have originated from the CBD source itself, the general lack of other minor cannabinoids (CBG, CBN) suggests that most samples presented via conversion from CBD isolate (which contains negligible amounts of  *cis*-∆^9^ THC). The more likely scenario is that the *cis*-∆^9^ THC present in converted samples arises from the racemization of the chiral center 6a due to isomerization of the 6-12 double bond after protonation (Figure 6). This hypothesis is supported by the observed presence in our analysis of cis-∆^9^ in samples that contain some conversion to ∆^10^ THC, as an analogous isomerization process with the endocyclic double bond would also racemize carbon 10a and lead to the formation of (+)-*cis*-∆^9^ THC. Further experiments will focus on the separation of the two cis isomers to verify that the (-)-*cis*-∆^9^ THC isomer is being formed during the conversion, as well as the presence of Intermediate A, to provide support for Pathway 2 as a likely route in the formation of *cis*-∆^9^ THC.


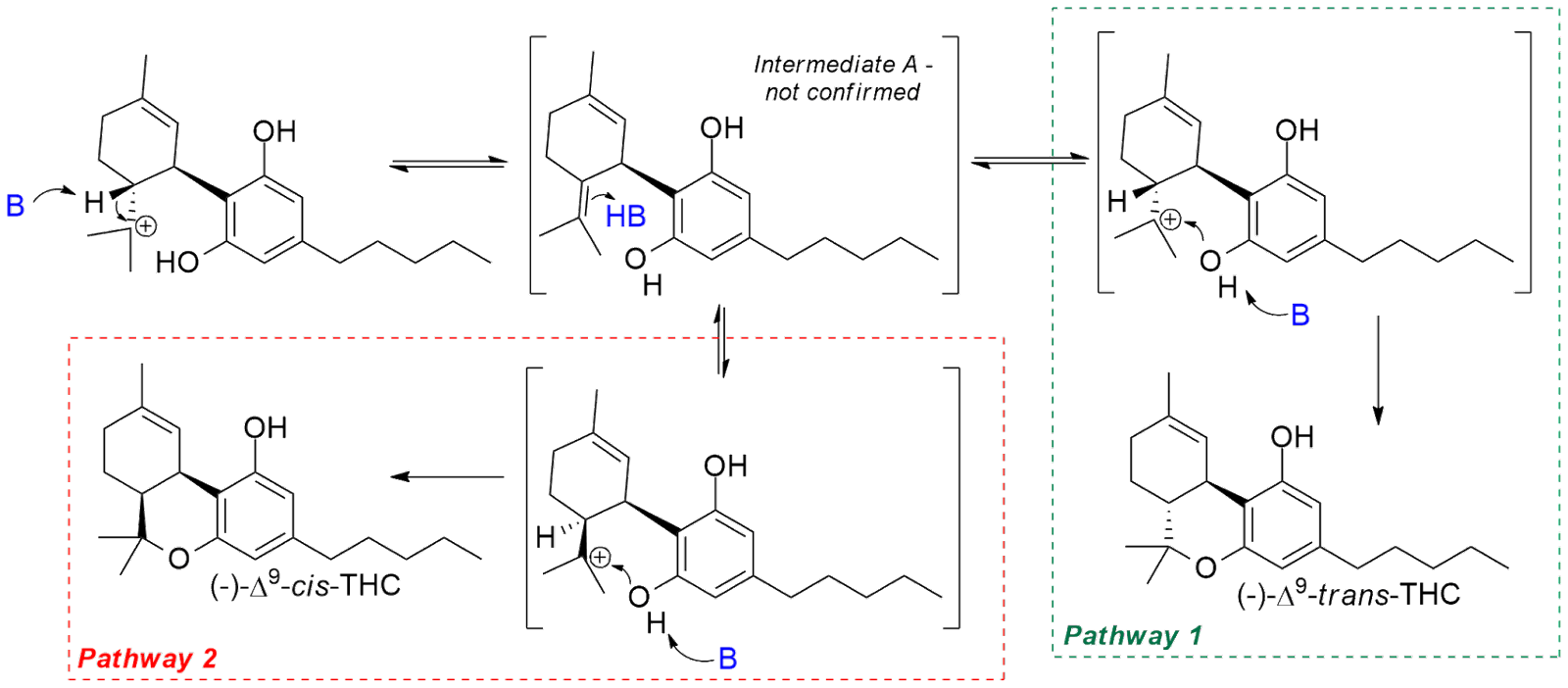


**Figure 6.** Racemization of chiral center at carbon 6a

The presence and relative quantity of (±)-*cis*-∆^9^ THC itself in natural hemp extracts, as well as in conversion reactions from CBD, can be a straightforward indicator of the source of ∆^9^ THC in concentrates and products. Since it is possible to form (-)-*trans*-∆^9^ THC directly from (-)-*trans*-CBD through an isomerization-free mechanism (Figure 1) as well as some contribution post-isomerization (Pathway 1, Figure 6), the ratios of trans:cis-∆^9^ in converted distillate far exceed the ratios seen in natural hemp extracts.

The insights gleaned from the analysis of distillates can be applied to products like the ∆^9^ THC edibles tested in this study, although there are challenges since the absolute concentrations of THC are different in the products. Nevertheless, the following two questions can be used as a starting point to categorize the ∆^9^ THC source in distillates and products: 1) Is there a clear and quantifiable amount of *cis*-∆^9^ THC in the product? 2) If yes, does the trans:cis ratio of ∆^9^ THC exceed 8:1? A negative answer to the first question suggests the product incorporates cannabis-derived distillate. A positive answer to the first question with a negative answer to the second question implies that the THC in the product is naturally derived from hemp without conversion. A positive answer to both questions suggests conversion from CBD is the likely source of the ∆^9^ THC in the product.

In addition to *cis*-∆^9^ THC as a metric for determination of THC source, levels of two other features in the UHPLC-DAD chromatogram appeared to strongly correlate with the origin of THC in distillate samples: CBG and the signal in the ∆^8^ THC region. Figure 7 displays an overlaid chromatogram of cannabis-derived ∆^9^ THC (black) and converted ∆^9^ THC (blue), normalized to the ∆^9^ THC content. In the left region of the spectrum, the CBG signal is clearly larger for cannabis-derived distillate. The large majority of cannabis-derived distillate in our analysis contained between 2-4% CBG (average 3%), while the average for CBG in distillate classified as converted was around ten times less, at 0.3%. The relative absence of CBG (and other minors) in converted samples could potentially be attributed to the presumed starting material for conversion: in most cases, CBD isolate (which contains small to negligible amounts of other cannabinoids) is likely used in order to maximize the amount of ∆^9^ THC formed after the reaction.


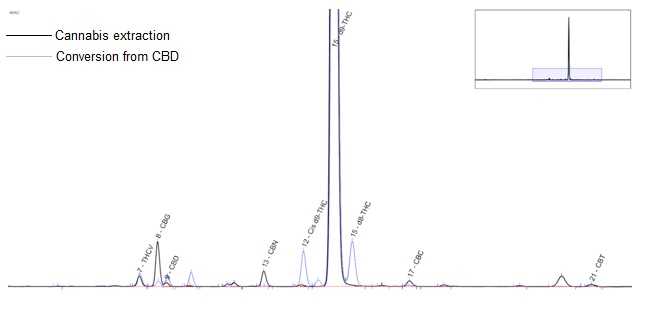


**Figure 7.** Overlay of chromatograms for ∆^9^ THC sourced from cannabis extraction and conversion from CBD.

To the left of the (*trans*)-∆^9^ THC signal in Figure 7 is the *cis*-∆^9^ THC signal, which can be seen to be present in the converted sample but essentially absent in the cannabis distillate. The signal just to the right of ∆^9^ THC is also absent in the cannabis distillate and is present and assigned to ∆^8^ THC in the converted sample. While the retention time and UV profile are an essentially identical match to an authentic ∆^8^-THC standard using our standard chromatographic conditions, variation of the column and flow rate causes the signal to split into two separate signals. One aligns with the ∆^8^-THC standard, while the other matches the retention time of the ∆^8^-iso-THC standard from Cayman Chemical (Figure 8, top center). ∆^8^-iso-THC is a product that results from acid-catalyzed cyclization of the phenolic-OH with the 9-10 double bond instead of the 6-12 double bond. ∆^8^-iso-THC has also been observed in conversions of CBD to ∆^8^-THC, along with its isomerized partner ∆^4,8^-iso-THC [10]. Conditions for forming ∆^9^-THC selectively require the favorability of cyclization over double-bond isomerization, so ∆^4,8^-iso-THC is not observed, and the isomerized ∆^8^-THC formation is limited, while the formation of cyclized but non-isomerized products ∆^9^-THC and ∆^8^-iso-THC are present.


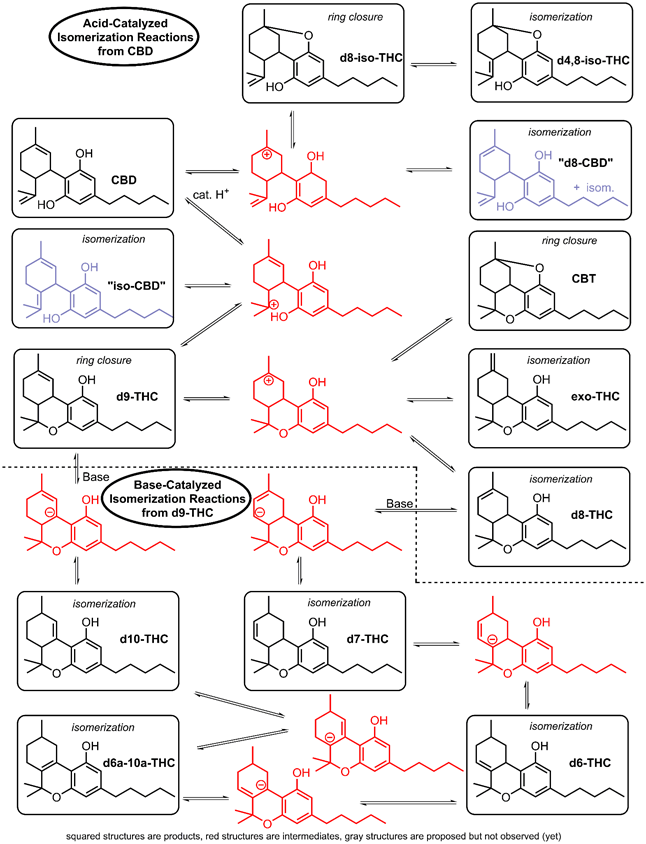


**Figure 8.** Reaction schemes for acid-catalyzed isomerization of CBD

This observation is much clearer by GC-MS, where ∆^8^-iso-THC and ∆^8^-THC exhibit much more facile separation (Figure 9). The overlaid chromatograms display ∆^8^-THC and ∆^9^-THC standards in blue and black, respectively, as well as a converted ∆^9^-THC sample in green. The converted sample displays a large ∆^9^-THC signal and a minor ∆^8^-THC signal; by comparison, the ∆^8^-iso-THC signal (second from left) is considerably larger.


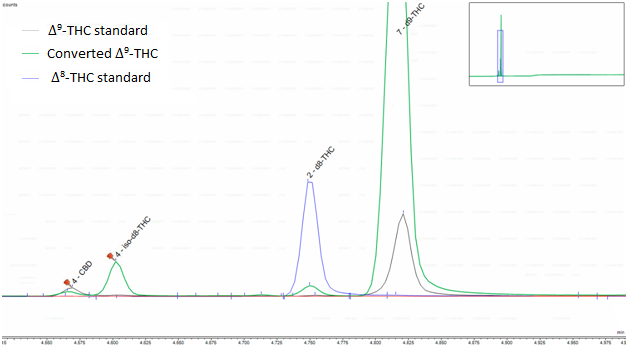


**Figure 9.** Overlay of GC-MS chromatograms for ∆^9^-THC standard, ∆^8^-THC standard, and a converted ∆^9^-THC sample.

Delineation of the ∆^8^-iso-THC and ∆^8^-THC amounts was not performed for this study, as the samples were run under standard UHPLC-DAD conditions for quantitation. The reported amounts of ∆^8^-THC, therefore, represent the combined contributions of both compounds. More importantly, the presence of one or both compounds, in addition to *cis*-∆^9^ THC, illustrates the limitations of selectivity in the conversion process from CBD to ∆^9^-THC.

Based on previous observations and other data [8, 19], metrics that provided excellent predictive power for high-THC distillate were set at 1% CBG, 0.25% *cis*-∆^9^-THC, and 1% ∆^8^-iso-THC + ∆^8^-THC. The 3-metric profile for cannabis-derived distillate is >1% CBG, <0.25% *cis*-∆^9^-THC, and <1% ∆^8^-iso-THC +∆^8^-THC. The profile for natural hemp-derived distillate is >1% CBG, >0.25% *cis*-∆^9^-THC, and <1% ∆^8^-iso-THC + ∆^8^-THC, with a trans:cis ∆^9^-THC ratio of <8:1. The profile for converted ∆^9^-THC distillate is <1% CBG, >0.25% *cis*-∆^9^-THC, and >1% ∆^8^-iso-THC + ∆^8^-THC, with a trans:cis ∆^9^-THC ratio of >8:1. 

Exact translation of fixed metrics for the ∆^9^-THC edible products in the study was not possible due to the wide range of ∆^9^-THC quantity in each product, but using the relative amounts of the three components along with some judgment calls, allowed for grouping of each product into the three categories with reasonable confidence.
